# Beyond the Bench: Research Helps Clean Up A Water Supply

**DOI:** 10.1289/ehp.115-a134

**Published:** 2007-03

**Authors:** Tanya Tillett

Many of the conveniences of modern life are made possible with man-made compounds. One such chemical, perfluorooctanoic acid (PFOA), has a broad spectrum of use, from the manufacture of non-stick cookware to aerospace technology. PFOA’s persistence in the environment is troubling, especially given studies demonstrating that exposure to the compound can cause developmental delays and cancer in lab animals. Thus, when PFOA was detected in the water supply of Little Hocking, a village located across the Ohio River from and downwind of a Washington, West Virginia, fluoropolymer manufacturing facility, researchers at the University of Pennsylvania NIEHS Center of Excellence in Environmental Toxicology (CEET) felt compelled to investigate. The contamination was first reported to Hong Zhang, a local doctor enrolled in a practicum residency for physicians in occupational and environmental medicine at the university.

According to CEET deputy director Edward Emmett, who also directs the center’s Community Outreach and Education Core, the research team’s immediate focus was on determining whether, how, and to what extent Little Hocking residents were being exposed to PFOA. The CEET investigators joined with community partners Grand Central Family Medicine in Parkersburg, West Virginia, and the Decatur Community Association in Cutler, Ohio, to design a study, recruit study participants, and collect data. The group applied for and received an environmental justice grant from the NIEHS, and began work in July 2004.

The investigators distributed questionnaires to a random sampling of residents who used either private or public drinking water sources, and examined blood serum samples to assess PFOA concentrations. PFOA water concentrations were obtained from the Ohio EPA. Levels averaged 3.55 ng/mL in 2002–2005, some of the highest ever reported in public water supplies in the United States.

Overall, blood serum analysis showed that the residents’ levels were 60–75 times higher than in the general U.S. population. The investigators found that serum PFOA was especially high in those who ate more home-grown fruits and vegetables. Emmett says it is unclear if this was due to PFOA making its way into the fruits and vegetables themselves, or to PFOA in water used for cooking, canning, and cleaning.

An air dispersion model based on estimated emissions from the Washington plant revealed that serum PFOA levels were no different for those people living in areas with higher air concentrations than for those living where there was minimal PFOA in the air. Regardless of location, higher concentrations were found in young children and older adults, as well as in people who worked directly with PFOA in production areas of the Washington plant (all three groups’ serum levels were almost twice as high as other residents accessing the Little Hocking water supply).

The research team examined all blood samples for biomarkers indicating DNA damage, but found no sign of adverse health effects. However, given what is known about the chemical’s effects in lab animals, Emmett says that lowering the Little Hocking residents’ exposure was prudent, and the independent research conducted by the partnership helped empower the community to secure a cleaner drinking water supply. “What has been so compelling and gratifying about this work has been witnessing how powerful credible, nonbiased information collected without conflict of interest can be in altering peoples’ behavior voluntarily,” says Emmett.

The Washington plant began offering bottled water to all residents being serviced in the Little Hocking Water District within days of an October 2005 community meeting where study results were presented. Other findings from the study suggested that carbon filters in the home could help to remove some PFOA from the water. Still, these were not considered viable long-term solutions, so a new filtration facility is being created to remove all PFOA from the water supply. The facility should be functioning in a few months.

Emmett says it is also gratifying to know that the partnership’s research has had a positive impact on the community. One resident remarked, “There was a large fine from EPA. There was a lawsuit, and a lot of money changed hands. But it’s [the CEET] study that has changed the water I drink.”

The study, which won first prize in the May 2006 EPA Science Forum, was described in two articles in the August 2006 *Journal of Occupational and Environmental Medicine*. A follow-up study is now under way to measure community members’ current PFOA blood levels. More information is available at http://lhwc8study.org/.

## Figures and Tables

**Figure:**
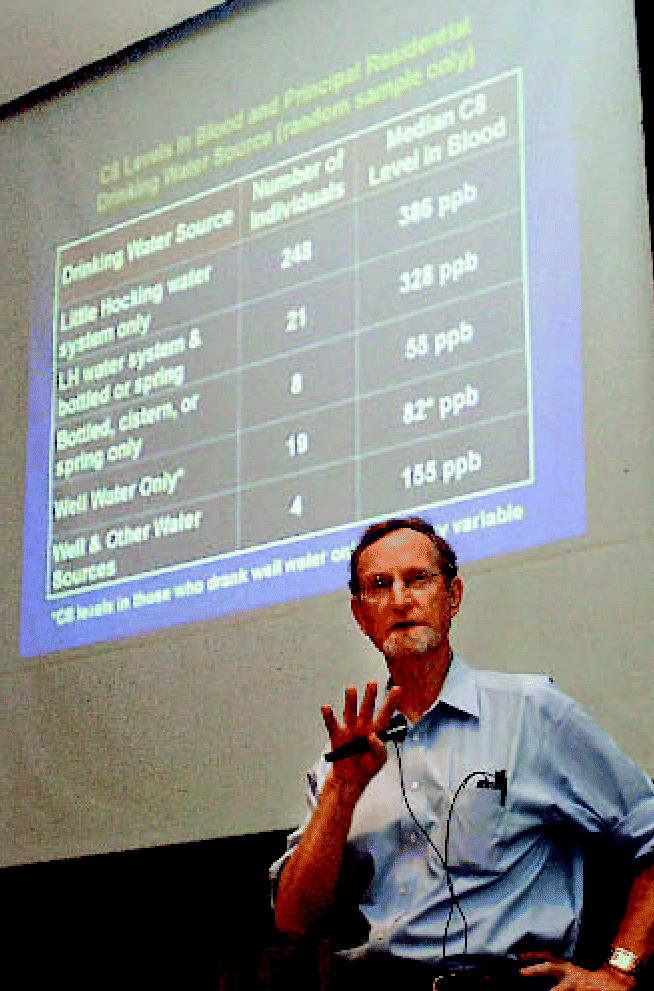
Town meeting Edward Emmett presents results of the CEET’s findings to the Little Hocking community.

